# Mammalian target of rapamycin inhibition protects glioma cells from temozolomide-induced cell death

**DOI:** 10.1038/s41420-023-01779-2

**Published:** 2024-01-05

**Authors:** Benedikt Sauer, Nadja I. Lorenz, Iris Divé, Kevin Klann, Anna-Luisa Luger, Hans Urban, Jan-Hendrik Schröder, Joachim P. Steinbach, Christian Münch, Michael W. Ronellenfitsch

**Affiliations:** 1 Dr. Senckenberg Institute of Neurooncology, University Hospital Frankfurt, Goethe University, Frankfurt am Main, Germany; 2 University Cancer Center Frankfurt (UCT), Frankfurt am Main, Germany; 3 German Cancer Consortium (DKTK), Partner Site Frankfurt/Mainz, a partnership between DKFZ and University Hospital Frankfurt, Frankfurt am Main, Germany; 4 Frankfurt Cancer Institute (FCI), University Hospital Frankfurt, Goethe University, Frankfurt am Main, Germany; 5 Institute of Biochemistry II, Goethe University, Frankfurt am Main, Germany; 6 Cardio-Pulmonary Institute, Frankfurt am Main, Germany

**Keywords:** CNS cancer, Cancer microenvironment

## Abstract

Glioblastoma is an incurable brain tumor with a median survival below two years. Trials investigating targeted therapy with inhibitors of the kinase mTOR have produced ambiguous results. Especially combination of mTOR inhibition with standard temozolomide radiochemotherapy has resulted in reduced survival in a phase II clinical trial. To date, this phenomenon is only poorly understood. To recreate the therapeutic setting in vitro, we exposed glioblastoma cell lines to co-treatment with rapamycin and temozolomide and assessed cell viability, DNA damage and reactive oxygen species. Additionally, we employed a novel translatomic based mass spectrometry approach (“mePROD”) to analyze acute changes in translated proteins. mTOR inhibition with rapamycin protected glioblastoma cells from temozolomide toxicity. Following co-treatment of temozolomide with rapamycin, an increased translation of reactive oxygen species (ROS)-detoxifying proteins was detected by mass spectrometry. This was accompanied by improved ROS-homeostasis and reduced DNA damage. Additionally, rapamycin induced the expression of the DNA repair enzyme O-6-methylguanine-DNA methyltransferase (MGMT) in glioblastoma cells with an unmethylated MGMT gene promotor. Inhibition of mTOR antagonized the cytotoxic effects of temozolomide in vitro. The induction of antioxidant defences and MGMT are two underlying candidate mechanisms. Further functional experiments in vitro and in vivo are warranted to characterize this effect that appears relevant for combinatorial therapeutic strategies.

## Background

With a median survival of less than two years with current treatment approaches, glioblastoma (GB) is a major focus of neurooncology research [1, 2]. Treatment concepts are limited by drug-induced toxicities as well as primary and acquired therapy resistance of tumors. The established first line treatment regimen for GB includes surgical resection followed by radiotherapy and temozolomide chemotherapy [3]. Temozolomide is hydrolyzed to 5-(3-methyl)1-triazen-1-yl-imidazole-4-carboxamide (MTIC) under physiological pH conditions which spontaneously forms the alkylating methyldiazonium cation [4]. Methylation of DNA molecules at N7 and O6 positions of guanine and N3 position of adenine bases is believed to be a major mechanism of cytotoxicity because the attempt to excise the modified nucleotide generates single- and double-strand breaks in the DNA that eventually lead to activation of apoptotic programs [5]. Exposure to temozolomide, however, also has been shown to increase ROS production which likewise is associated with DNA damage and therefore represents an additional cause of genomic damage [6–8]. The methylation status of the promotor of the MGMT gene regulates transcription and expression of this DNA damage repair enzyme and determines effectiveness of temozolomide treatment [9]. Sufficient dosage of classical chemotherapies like temozolomide can be problematic and limited by hematological toxicities which is associated with reduced survival at least in patient subgroups [10]. Therefore, novel and tumor-selective therapeutics ideally with a mild or at least different toxicity profile from classical chemotherapeutics are urgently needed.

Genetic analyses have confirmed epidermal growth factor receptor (EGFR)-dependent signaling as one of the most commonly altered signaling networks in GB [11]. With regard to EGFR, more than 45% of GB show genetic alterations (amplification or mutation), additionally mutations of the downstream signaling suppressor PTEN occur in more than 35% of tumors [11]. One major downstream signaling node of EGFR is the mammalian (or synonymous: mechanistic) target of rapamycin (mTOR), a serine/threonine kinase that is found in two different mTOR multiprotein complexes (mTORC) 1 and 2. mTORC1 is a major regulator of cellular growth and protein translation. The two best characterized phosphorylation targets are S6 kinase 1 (S6K1) and eukaryotic translation initiation factor 4E (eIF4E) binding protein 1 (4EBP1) both implicated in the regulation of mRNA translation. mTORC2 has been identified as an activator of Akt via phosphorylation at Ser473 [12]. Akt signaling has long been implicated in cancer progression and development. First generation mTOR inhibitors include rapamycin as the original compound and its derivatives everolimus and temsirolimus. Everolimus has been approved for the treatment of advanced renal cancer, neuroendocrine tumors of pancreatic origin as well as hormone receptor positive advanced breast cancer [13–15]. First generation mTOR inhibitors have however been found to not completely inhibit mTORC1 [16]. With the discovery of mTORC2, several new ATP competitive inhibitors have been developed with superior mTORC1 inhibition as wells as also inhibition of mTORC2. Torin1 is one such compound specifically targeting both mTORC 1&2 [17]. Torin2 is a slightly modified version of the original compound torin1 to improve pharmacokinetics and has been shown to cause growth inhibition in cancer cells as well as shown efficacy in a KRAS-driven lung cancer model in combination with the mitogen-activated protein/extracellular signal-regulated kinase (MEK) inhibitor AZD6244 [18]. Because mTOR is a component of frequently activated signaling cascades in GBs and plausible therapeutic target, clinical trials to evaluate mTOR inhibitors for treatment are underway. Thus far, therapeutic benefit has been suggested in GB subgroups with activated mTOR signaling based on immunohistochemistry analyses from clinical trials without temozolomide, e.g. the EORTC 26082 trial [19]. In the large randomized RTOG0913 phase II trial that included 171 patients with newly diagnosed GB, addition of the mTORC1 inhibitor everolimus to standard temozolomide radiochemotherapy was however associated with reduced overall survival as well as increased toxicity [20]. Compensatory activation of Akt via unregulated mTOR complex C2 signaling was discussed as a possible factor, however no clear explanation of the detrimental effect of combination therapy has been found so far.

In this experimental study we investigated the implications of mTOR inhibition in the context of temozolomide therapy as the major chemotherapeutic agent in GB therapy and discovered a cytoprotective effect of mTOR inhibition. Employing the novel mass spectrometry-based translatomic analysis mePROD [21] we found an increased translation of ROS-detoxifying proteins under rapamycin treatment in glioblastoma cell lines. In cell lines with an unmethylated MGMT promotor, rapamycin increased levels of MGMT expression. This data provides a potential explanation for the antagonistic effects of mTOR-Inhibition during temozolomide treatment.

## Results

### mTOR inhibition protects glioma cells from temozolomide toxicity

**Fig. 1:**
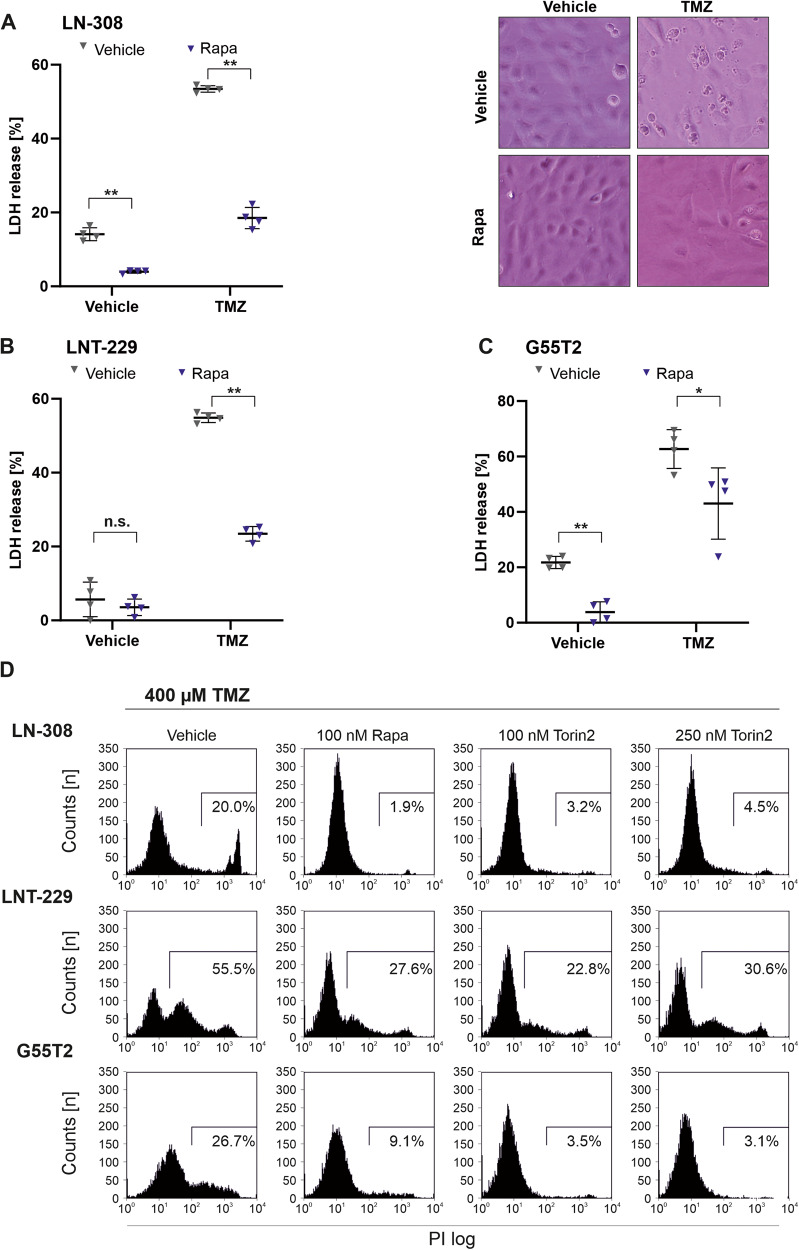
MTOR inhibition protects human glioma cells from temozolomide-induced cell death. **A**–**C** LN-308, LNT-229 or G55T2 cells were incubated in serum-free DMEM for 3 days with vehicle or 100 nM rapamycin with or without 400 µM temozolomide. Cell death was quantified by LDH-release (*n* = 4, mean ± SD; n.s. not significant, *p < 0.05, **p < 0.01 Student’s *t*-test). Representative photographs of LN-308 cells are included in the right-hand panels (bright field microscopy, ×48 magnification) **D** LN-308, LNT-229 or G55T2 cells were incubated in serum-free DMEM for 3 days with 400 µM temozolomide in the presence of 100 nM rapamycin or 100 or 250 nM torin2. Cell death was assessed by PI staining (*n* = 3, representative results are shown). The FACS gate defines cells considered PI positive.

Temozolomide is the major chemotherapeutic option for GB therapy and glioma cells differ in their sensitivity to acute temozolomide toxicity. We have previously shown that mTOR inhibition renders LNT-229 glioma cells more resistant towards CCNU as well as vincristin chemotherapy [16]. To test whether mTOR inhibition affects the effectiveness of temozolomide chemotherapy in glioblastoma cells, LN-308, LNT-229 and G55T2 cells were treated with temozolomide in the presence of rapamycin or torin2. mTOR inhibition protected LN-308 cells from temozolomide as indicated by increased cell density (Supplementary Fig. 1A) and reduced LDH release in the presence of rapamycin or torin2 (Fig. 1A, left panel). This effect also caused LN-308 cells to retain an almost normal morphology when treated with rapamycin and temozolomide in comparison to temozolomide alone (Fig. 1A, right panel). Similar results were obtained for LNT-229 as well as G55T2 cells (Fig. 1B, C). To further validate the reduction in temozolomide-mediated cell death in glioma cells, LN-308, LNT-229 as well as G55T2 cells were again exposed to temozolomide and mTOR inhibitors. Using propidium iodide staining, reduced temozolomide cytotoxicity under mTOR inhibition was confirmed (Fig. 1D, Supplementary Fig. 1B). To validate this effect under lower temozolomide concentrations, analyses of clonogenic survival were performed in LNT-229 and LN-308 cells. Similarly, temozolomide treatment resulted in reduced clonal survival, which could be relieved by co-treatment with rapamycin (Supplementary Fig. 1C).

### Co-treatment of temozolomide and rapamycin leads to increased translation of anti-apoptotic and ROS-detoxifying enzymes

**Fig. 2:**
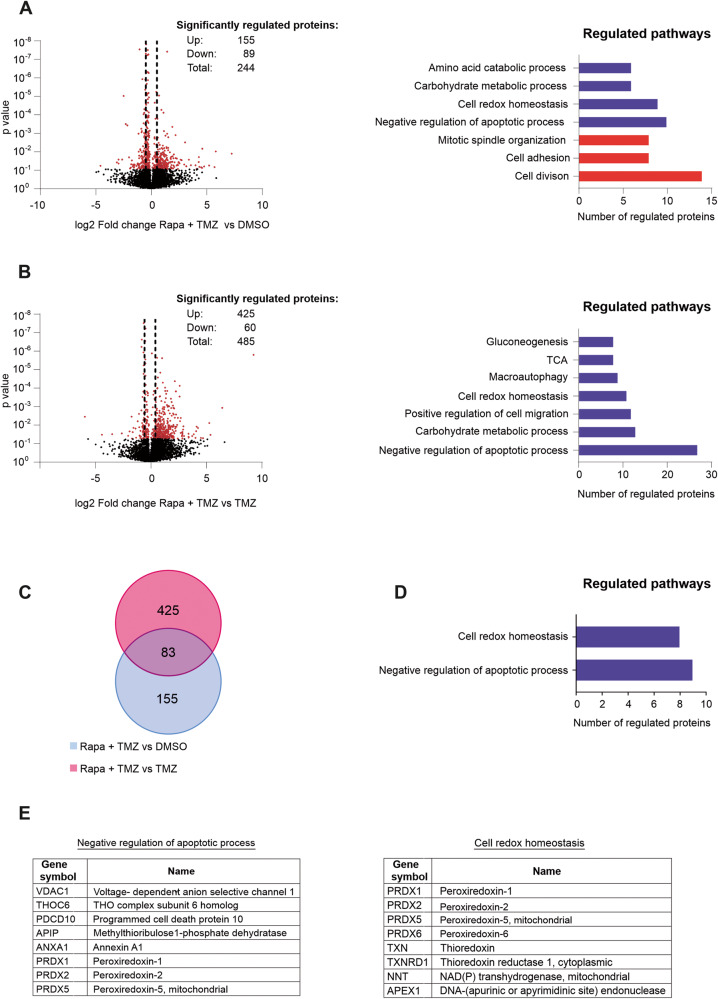
Combination of temozolomide and rapamycin leads to increased translation of anti-apoptotic and ROS-detoxifying enzymes. Changes in cellular translatome following co-therapy with temozolomide and rapamycin. LN-308 cells were incubated with DMSO, rapamycin, temozolomide or with a combination of rapamycin and temozolomide in DMEM for SILAC containing 100 μg/mL Arg10, 100 μg/mL Lys8 for 6 h. **A** left panel: Volcano plot of fold changes versus *p* values between DMSO and combination of temozolomide and rapamycin. Positive values display upregulated proteins in the combination group. The dashed line indicates a fold change cutoff of 0.5 increase or decrease in translation, the red dots indicate *p* values < 0.05. Right panel: Bar chart of proteins significantly upregulated (blue) and downregulated (red) that cluster for gene ontology (GO). **B** left panel: Volcano plot fold of fold changes versus *p* values between temozolomide monotherapy and combination of temozolomide and rapamycin. Positive values display upregulated proteins in the combination group. The dashed line indicates a fold change cutoff of 0.5, the red dots indicate *p* values < 0.05. Right panel: Bar chart showing the proteins significantly upregulated (blue) and downregulated (red) that cluster for the respective gene ontology (GO) terms. **C** Venn diagram of proteins significantly upregulated in combination therapy vs. DMSO or temozolomide monotherapy. **D** Bar chart of overlapping proteins significantly upregulated that cluster for the respective GO terms. **E** List of proteins corresponding to the GO terms that were detected in mass spectrometry analyses .

To further examine the molecular basis of the protective effect of mTOR inhibition towards temozolomide toxicity, we analyzed changes of the cellular translatome, employing the novel method mePROD [21], following 6 h treatment with rapamycin, temozolomide and the combination of both agents. The strength of this method is its high sensitivity to detect even low levels of newly translated proteins against the background of the static cellular proteome, thereby allowing detection of acute changes in translational programs. Altogether, 6684 proteins were identified. We chose a log_2_ fold change of 0.5 and −0.5 and *p* < 0.05 as cutoffs for significance. A list of the identified proteins and the respective differential expression is provided in the supplement (Supplementary table 1). Comparing the combination of rapamycin and temozolomide with the vehicle condition, 244 proteins were regulated significantly with 155 up- and 89 downregulated proteins (Fig. 2A). Using the DAVID bioinformatics analysis tool for pathway enrichment analysis we identified cell division and adhesion, as well as mitotic spindle organization as the major downregulated pathways, whereas negative regulation of apoptosis, cell redox homeostasis and metabolism of carbohydrates and aminoacids made up for the most upregulated pathways (Fig. 2A). Interestingly, treatment with rapamycin alone led to a highly distinct profile of newly translated proteins, with downregulation of proteins involved in signal transduction (MAP-kinase and protein kinase B) and upregulation of RNA-processing and nonsense-mediated-decay which has been described previously in other cell types [22] (Supplementary Fig. 2A). Monotherapy with temozolomide led to a decreased global translation with downregulation of 225 of 264 total significantly regulated proteins. Like in the combination therapy, cell division and cell adhesion were among the most regulated pathways and can thus most likely be attributed to the effect of temozolomide (Supplementary Fig. 2B). Next, to better understand the effect of rapamycin in the context of combination therapy, we compared the translatome of the temozolomide treated cells with the combination of rapamycin and temozolomide. Here, we detected an accentuated increase in translation with 425 of 485 proteins being upregulated. Similar to the comparison with DMSO, negative regulation of apoptosis and cell redox homeostasis were among the strongest regulated pathways (Fig. 2B). To optimally extract the effect of rapamycin, we scanned the profiles of both groups (DMSO vs rapamycin + temozolomide and temozolomide vs rapamycin + temozolomide) for overlapping proteins. In total, 83 proteins were identified (Fig. 2C). Pathway analysis of these 83 proteins contained in both groups again revealed negative regulation of apoptosis and cell redox homeostasis as the two main pathways (Fig. 2D) with a corresponding spectrum of regulated proteins (Fig. 2E). Of note, the family of peroxiredoxins accounted for a major part of the upregulated proteins in the combinatory treatment. The six isoforms are well characterized mediators of cellular ROS homeostasis and have been shown to be upregulated in -several tumor entities as well as linked to unfavorable prognosis and therapy resistance [23–26]. We concluded that the effect of rapamycin highly differs depending on the presence of other stressors like temozolomide and could help glioma cells to survive by enhancing the cellular defense against ROS.

### Temozolomide induces ROS in human GB cell lines

**Fig. 3:**
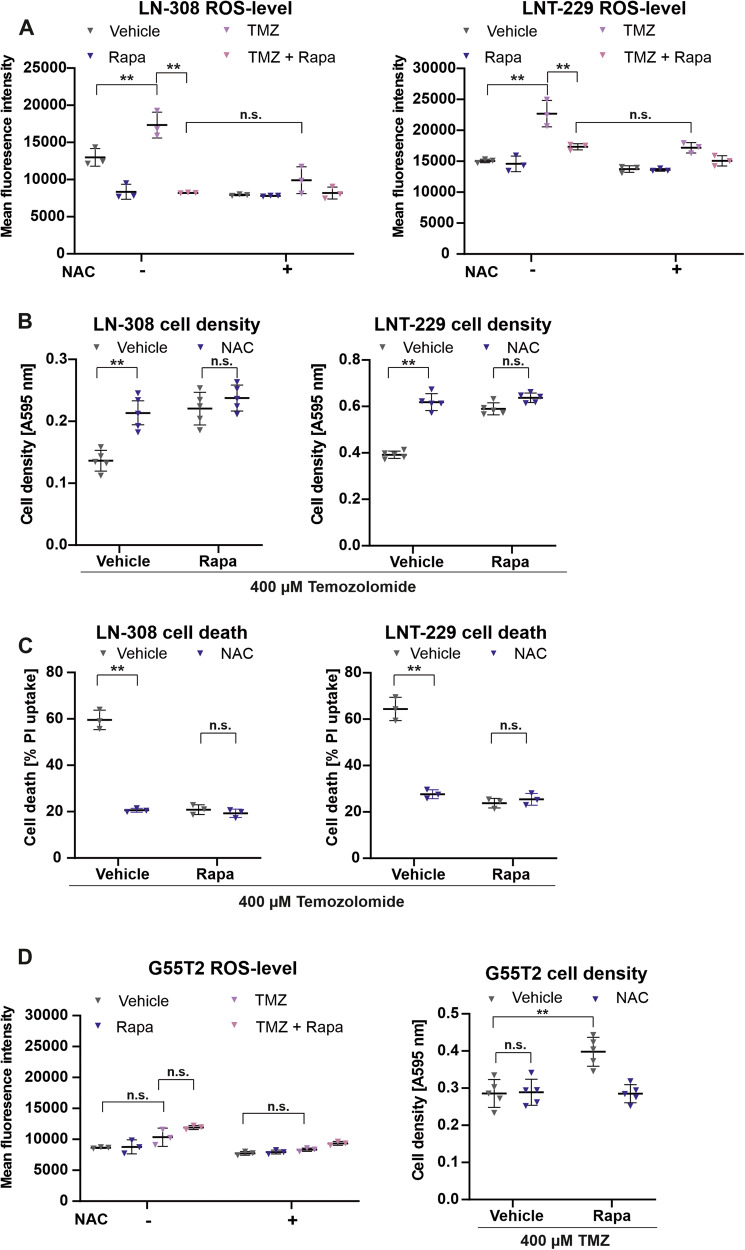
MTOR inhibition attenuates temozolomide-induced reactive oxygen species. **A** LNT-299, LN-308 cells were incubated serum-free DMEM for 6 h in the presence of 400 µM temozolomide, 100 nM rapamycin or combinatory treatment with and without the addition of 1 mM NAC. ROS levels were measured by H2DCFDA-FACS (*n* = 3, mean ± SD, *p < 0.05 Student’s *t*-test). **B** LNT-299, LN-308 cells were incubated under the conditions stated above for 3 days. Cell density was assessed by crystal violet (CV) staining (*n* = 5, **p < 0.01 Student’s *t*-test). **C** LNT-299, LN-308 cells were incubated under the conditions stated above for 3 days. Cell death was assessed by PI staining (*n* = 3, mean ± SD, *p < 0.05 Student’s *t*-test). **D** G55T2 cells were incubated under the conditions stated above for 6 h (left panel) and for 3 days (right panel). ROS levels were measured by H2DCFDA-FACS (left panel), cell density was assessed by CV staining (right panel) *(n* = 3, **p < 0.01 Student’s *t*-test).

To determine whether the rapamycin-mediated protective effects against temozolomide might be due to changes in redox homeostasis, we measured levels of intracellular ROS. Indeed, treatment with temozolomide increased ROS- levels in LN-308 and LNT-229 cells while co-treatment with rapamycin—in accordance with our translatomic data – reduced the amount of ROS to the level of the vehicle condition in LNT-299 cells and even below the level of vehicle in LN-308 cells (Fig. 3A). To compare rapamycin with an established ROS-inhibitor, we additionally treated the cells with N-acetylcysteine (NAC). NAC serves as a scavenger of radicals and prodrug for the antioxidant glutathione [27]. The reduction of temozolomide-induced ROS by NAC matched the effect of rapamycin, while combination of NAC and rapamycin had no significant additional effect. To test whether NAC has the same effect on cell density as rapamycin when combined with temozolomide, we treated LN-308 and LNT-229 cells with temozolomide, rapamycin in presence and absence of NAC. Cells treated with rapamycin or NAC displayed a higher and very similar cell density cell density after 72 h while again the combination had no significant beneficial effect. (Fig. 3B). This is also reflected by a comparable degree of protection against temozolomide-induced cell death (Fig. 3C). Surprisingly, in G55T2 cells while temozolomide did induce ROS, only NAC and not rapamycin was able to reduce ROS levels (Fig. 3D). With regard to the control conditions, treatment with rapamycin alone had a growth inhibitory effect only in LN-308 cells and neither cell line displayed relevant cell death upon rapamycin treatment (Supplementary Fig. 3). To test whether further elevated levels of ROS would mitigate the protective effect of rapamycin, we employed buthionine sulfoximine (BSO) an established inductor of cellular ROS that acts via the inhibition of glutathione synthesis [28]. Adding BSO to the combination treatment of temozolomide and rapamycin, abrogated the protective effect of rapamycin and resulted in comparable cell densities as well as comparable amounts of cell death between the temozolomide and the combination therapy group of temozolomide and rapamycin. Of note, BSO treatment alone already caused a mild induction of cell death (Supplementary Fig. 4A, B).

### Rapamycin increases MGMT levels in MGMT promotor unmethylated glioma cells

**Fig. 4:**
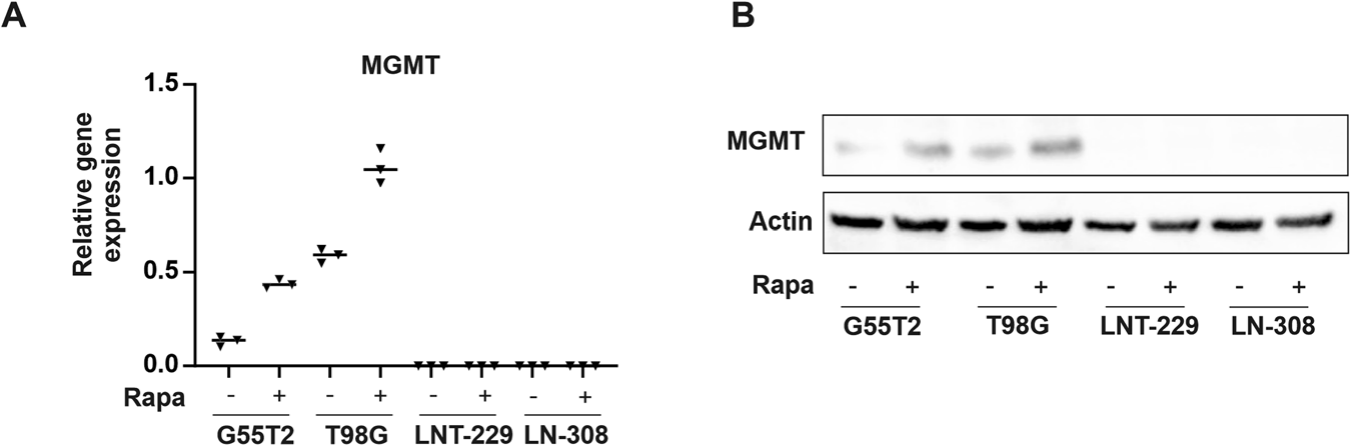
MTOR inhibition induces MGMT transcription and protein in MGMT gene promotor unmethylated glioblastoma cells. LNT-299, LN-308, T98G and G55T2 cells were incubated with 100 nM rapamycin for 24 h in serum-free medium. **A** Gene expression of MGMT was quantified (*n* = 3, mean ± SD, *p < 0.05, **p < 0.01, Student’s *t*-test). **B** Representative immunoblot displaying MGMT protein levels. Actin was used as loading control.

While rapamycin protected G55T2 cells from temozolomide (Fig. 1C, D), no effect of rapamycin on cellular ROS levels could be detected indicating a different mechanism of rapamycin-mediated resistance to temozolomide in this cell line. An important distinguishing factor between LNT-229, LN-308 and G55T2 cells is the methylation status of the MGMT promotor. In contrast to LNT-229 and LN-308 cells, G55T2 have an unmethylated MGMT promotor [29]. We therefore interrogated whether rapamycin altered the expression of MGMT in MGMT promotor unmethylated glioma cells thereby antagonizing temozolomide. Indeed, gene expression was significantly induced in the MGMT promotor unmethylated cell line G55T2 while no expression was detectable in the MGMT promotor methylated cell lines LN-308 and LNT-229 following treatment with rapamycin (Fig. 4A). To confirm an effect of rapamycin on MGMT gene expression in MGMT promotor unmethylated GB cells, we complemented experiments with T98G cells which yielded similar results (Fig. 4A). Consistently, protein levels of MGMT were increased following treatment with rapamycin (Fig. 4B). The whole membrane is supplied in Supplementary Fig. 2.

### Rapamycin impairs temozolomide-induced genomic damage

**Fig. 5:**
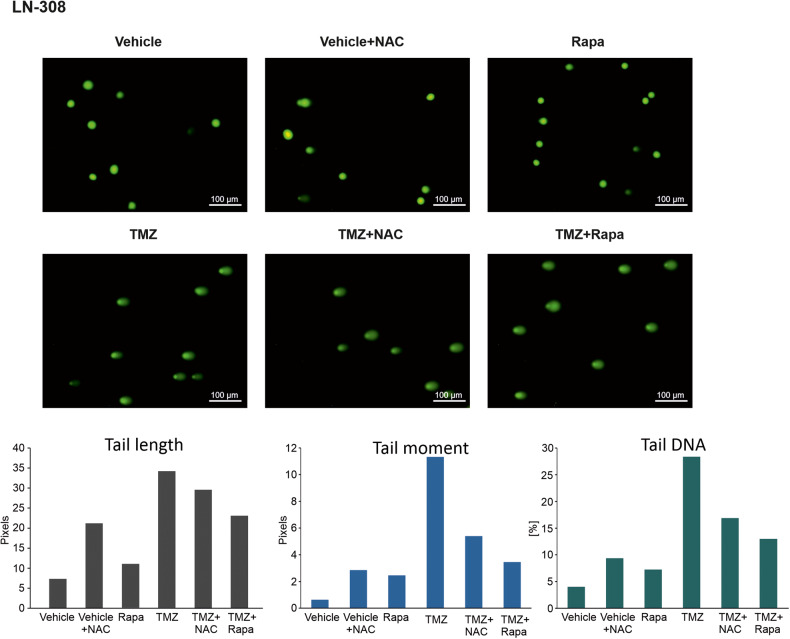
MTOR inhibition and N-acetylcysteine attenuate temozolomide-induced DNA fragmentation. Exemplary images and quantification of percentage of DNA in the tail, tail length and tail moment of alkaline comet assay of LN-308 cells treated with 400 µM temozolomide, rapamycin or the combination of both agents with and without the addition of 1 mM NAC.

In order to quantify temozolomide-induced DNA damage in context of mTOR inhibition, we performed a comet assay in LN-308 cells. We treated the cells with temozolomide, rapamycin and NAC as indicated and determined the percentage of DNA in the tail, tail length and tail moment (DNA in the tail multiplied by the distance between the means of the head and tail distributions). As expected, all parameters reflecting the amount of DNA damage were increased in temozolomide treated cells. Co-treatment with rapamycin as well as NAC reduced DNA damage inflicted by temozolomide, indicated by a reduction of percent DNA in the tail, tail length and tail moment which can most likely be attributed to the reduction of ROS-induced DNA damage (Fig. 5).

## Discussion

Since the introduction of temozolomide as standard chemotherapy for GB no new drugs have been approved. Inhibition of signal transduction is still a promising therapeutic approach, however combinatorial treatments may be necessary to achieve sufficient pathway inhibition. So far, however, none of the combination therapy trials have increased survival [16, 30] and some have even produced antagonistic results [20]. In our study, we found that the combination of mTOR inhibitors with temozolomide resulted in reduced cytotoxicity. This effect was detectable both with the mTORC1-specific inhibitor rapamycin as well as the dual mTORC1 and 2 inhibitor torin2 (Fig. 1). Apoptosis is considered the main cell death-program under temozolomide which induces single- and double-strand breaks [31, 32] and therefore most likely also accounts for a major portion of the cell death in our experiments. Analysis of the translatome of glioma cells under treatment with temozolomide and rapamycin revealed enhanced translation of ROS-detoxifying enzymes, especially peroxiredoxins. This family of antioxidant enzymes has often been associated with tumor progression and therapy resistance in various tumor entities like lung, breast and bladder cancer [23–26]. Our data suggests that addition of rapamycin attenuates genomic damage by restoring ROS homeostasis. While treatment with temozolomide led to an induction of ROS, a phenomenon that has previously been described [6–8], co-treatment with rapamycin significantly reduced temozolomide-induced ROS and was accompanied by reduced cell death. (Fig. 3A, C) Furthermore, DNA damage inflicted by temozolomide was decreased, probably due to decreased ROS-induced genotoxic stress (Fig. 5). These observations are in line with the notion of previously published articles, that enhanced antioxidative defenses promote resistance of cancer cells against alkylating agents like temozolomide [33, 34]. Interestingly, monotherapy with rapamycin did not alter translation of proteins involved in redox homeostasis as much as when combined with temozolomide (Supplementary Fig. 2A). It is plausible that the presence of an additional stressor is necessary for the observed effect to occur. Whether the response to other ROS inducing and DNA damaging therapeutic interventions, i.e., irradiation, is modulated by mTOR inhibition in a similar manner needs further investigation and might differ between different tissues and tumor entities. Similarly the molecular GB subgroup and specific pathway activation of the tumor tissue might influence the effect of mTOR inhibition on cell survival under temozolomide treatment. It is noteworthy that the definition of molecular GB subtypes is a dynamic field with more refined subtypes continuously evolving including a recently defined pathway-based classification [35]. We are not aware of any GB subtype-specific analyses of the RTOG0913 tumor samples which would be a very interesting aspect of future research. At least for the subgroups of MGMT gene promoter methylated and unmethylated GB tumors, our data suggests independent mechanisms of rapamycin-mediated therapy resistance (Fig. 4 A,B).

We have previously described the induction of the DNA damage-inducible transcript 4 (DDIT4) by various stressors including temozolomide [36]. Mechanistically, DDIT4 mediates the disinhibition of the tuberous sclerosis 1/2 (TSC1/TSC2) complex which itself is a major negative regulator of mTORC1 [37]. Similar to our results with pharmacological mTOR inhibition, physiological mTOR inhibition via DDIT4 had protective effects in regards to temozolomide toxicity [36]. Until now, the mechanism of this effect was unclear. In the light of our data, it seems plausible that DDIT4 affects ROS homeostasis in a similar way as rapamycin since both act via the inhibition of mTORC1. In MGMT promotor unmethylated glioma cells mTOR inhibition led to an induction of MGMT which may explain the protective effect despite the lack of effect on ROS levels in these cells. However, further investigation is necessary to define the implications of altered MGMT expression by mTOR inhibition.

In summary, our results indicate that co-administration of temozolomide chemotherapy and mTOR inhibitors reduces temozolomide efficacy via recovery of ROS homeostasis in MGMT promotor methylated glioma cells. This provides a potential explanation for the detrimental effect observed in patients treated with this combination therapy [20]. In future studies, further vadidation and comprehensive analysis of the underlying molecular mechanisms and key factors of mTOR inhibition-mediated ROS homeostasis as well as functional experiments are necessary. This is a challenging task, since the effects can most likely not be attributed to singular factors and rather depend on networks of interconnected pathways. Our data sheds light on the limitation of temozolomide chemotherapy in combination with mTOR inhibitors which can be a starting point for the design of new treatment schedules or combinations taking redox homeostasis into account.

## Materials and methods

### Reagents, cell lines and culture conditions

Rapamycin and torin2 were purchased from Tocris (Bristol, UK), all reagents not specified were purchased from Sigma (Taufkirchen, Germany). LNT-229 cells have been described [16]; G55T2 cells were a kind gift of Manfred Westphal and Katrin Lamszus (Hamburg, Germany) [38] and LN-308 cells were a kind gift of Dr. N de Tribolet (Lausanne, Switzerland) and recently have been validated by short tandem repeat (STR) profiling. Testing for mycoplasma contamination was performed once every month. Molecular characteristics of these cell lines have been described [29]. Cell lines were maintained in Dulbecco’s modified eagle medium (DMEM) (Gibco) containing 10% fetal calf serum (FCS) (Biochrom KG, Berlin, Germany), 100 IU/ml penicillin and 100 µg/ml streptomycin (Life Technologies, Karlsruhe, Germany) [16].

### Temozolomide treatment

Temozolomide concentrations were chosen based on the experimental setup. For the study of acute temozolomide toxicity higher doses (around 400 µM) are commonly employed [39, 40] and such concentrations were also used in ROS and proteomics experiments. For clonal survival analyses significantly lower doses around 10 µM temozolomide are sufficient [39, 41].

### Cell density and cell viability assays

Cell density was assessed by crystal violet (CV) staining as previously described [16, 42, 43]. For cell growth evaluation, 5000 cells were seeded per well of a 96 well plate. After incubation overnight in DMEM containing 10% FCS, the cells were incubated as indicated. Cell viability measurement using propidium iodide (PI) uptake was done by fluorescence activated cell sorting (FACS) analysis and has been described [16]. Cell viability analysis by lactate dehydrogenase (LDH) release assay was performed with the Cytotoxicity Detection Kit (LDH) (Roche, Mannheim, Germany) and has also been described [16, 44]. Cytotoxicity assays were performed in subconfluent cells in serum-free DMEM. For evaluation of clonal survival, 1000 cells per well were seeded in a 6-well plate. Cells were treated with vehicle, temozolomide, rapamycin and the combination of temozolomide and rapamycin in serum-free medium. Following a 24 h incubation, the treatment medium was exchanged with fresh DMEM containing 10% FCS. The experiment was stopped by CV staining once the clones neared close adjacency to neighboring clonal colonies. Subsequently, the clones were manually counted under a microscope.

### Determination of reactive oxygen species (ROS)

Cells were incubated for 6 h in serum-free medium containing serum-free medium and the indicated substances. This was followed by a wash step with PBS and incubation with 10 µM of the fluorescent dye dichlorodihydrofluorescein diacetate (H2DCFDA) for 30 min at 37°. Subsequently, fluorescence intensity was determined by flow cytometry (FACS, BD Canto II, BD Biosciences, San Jose, CA, USA) and analyzed using the BD FACS Diva software version 6.1.3 (BD Biosciences, Franklin Lakes, NJ, USA)

### RNA extraction and quantitative reverse transcription-PCR (qPCR) analysis

Quantitative PCR was performed as previously described [29]. Briefly, RNA was isolated using TRIzol® (Invitrogen, Karlsruhe, Germany) and the Total RNA Kit (Blirt, Gdansk, Poland). Complimentary DNA was synthesized using the Vilo cDNA synthesis kit (Invitrogen) according to the manufacturer’s protocol. PCR was performed using the IQ5 real-time PCR detection system (Biorad, Munich, Germany) with Absolute Blue Q-PCR Mastermix with SybrGreen+Fluorescein (Thermo Fisher Scientific, Hamburg, Germany) [29] Primer pairs used are displayed in Table 1.

**Table 1:** Primer pairs for qPCR.

Target gene	Forward sequence (5′ → 3′)	Reverse sequence (5′→ 3′)
18S	CGGCTACCACATCCAAGGAA	GCTGGAATTACCGCGGCT
SDHA	TGGGAACAAGAGGGCATCTG	CCACCACTGCATCAAATTCATG
O^6^ methylguanine-DNA methyltransferase (MGMT)	ACCGTTTGCGACTTGGTACTT	GGAGCTTTATTTCGTGCAGACC

### Lysate preparation and immunoblot analysis

For lysate generation, cells were washed with ice-cold phosphate-buffered saline (PBS) and immediately frozen in a layer of liquid nitrogen. Protein extraction was carried out using lysis buffer P consisting of 50 mM Tris-HCL (pH 8.0),120 mM NaCl, 5 mM EDTA, and 0.5% NP-40) with the addition of 1% Halt™ Protease and Phosphatase Inhibitor Single-Use Cocktail (Thermo Fisher Scientific, Hamburg, Germany). Protein concentration was determined by the Bradford method. Cell lysates were diluted in Laemmli buffer and separated by electrophoresis. This was followed by transfer (“wet blotting”) to a nitrocellulose membrane (0.45 µm; GE Healthcare, Little Chalfont, UK). Membranes were incubated overnight with the following primary antibodies: MGMT (#2739) (Cell Signaling Technology, Danvers, MA, USA) and Actin (#sc-1616) (Santa Cruz Biotechnology, Santa Cruz, CA, USA). Anti-goat antibodies were purchased from Santa Cruz Biotechnology (#sc-2020). The secondary anti-rabbit antibody was purchased from Jackson ImmunoResearch (#111-036-144; West Grove, PA, USA). A chemiluminescent solution consisting of 1 mL solution A (200 mL 0.1 M Tris-HCl pH 8.6, 50 mg luminol), 100 µL solution B (11 mg p-hydroxycoumaric acid, 10 mL DMSO), and 0.3 µL H_2_O_2_ (30%) was used for detection.

### Sample preparation for mass spectrometry

Cells were seeded in triplicates and incubated in DMEM for SILAC (ThermoFisherScientific) containing 100 μg/mL Arg10 (Cambridge Isotope Laboratories), 100 μg/mL Lys8 (Cambridge Isotope Laboratories) with the addition of DMSO, 400 µM temozolomide, 100 nm rapamycin or the combination of both substances for 6 h. Preparation of the samples was carried out as previously described [21]. In short, lysates were sonicated with Sonic Vibra Cell and precipitation of the lysates was performed using methanol/chloroform. Proteins were resuspended in 8 M Urea/10 mM EPPS pH 8.2 and protein concentration was measured by Bradford assay. The samples were then diluted to 2 M Urea with 10 mM EPPS pH 8.2 and incubated overnight with 1:50 LysC (Wako Chemicals, Neuss, Germany) and 1:100 Sequencing grade trypsin (Promega, Madison, WI, USA) for digestion. Digests were then acidified using TFA and purified by tC18 SepPak (50 mg, Waters, Milford, MA, USA). 25 µg peptides per sample was used for TMT-labeling and normalized after a single injection measurement by LC-MS/MS to equimolar ratios for each channel. Pooled peptides were dried for offline High pH Reverse phase fractionation by HPLC.

### Offline high pH reverse phase fractionation

Peptide fractionation was performed using a Dionex Ultimate 3000 analytical HPLC. 250 µg of pooled TMT-labeled samples were resuspended in 10 mM ammonium-bicarbonate (ABC), 5% ACN, and separated on a 250 mm long C18 column (X-Bridge, 4.6 mm ID, 3.5 µm particle size; Waters) using a multistep gradient from 100% Solvent A (5% ACN, 10 mM ABC in water) to 60% Solvent B (90% ACN, 10 mM ABC in water) over 70 min. Eluting peptides were collected every 45 s into a total of 96 fractions, which were cross-concatenated into 12 fractions and dried for further processing.

### Liquid chromatography mass spectrometry

All data was acquired in centroid mode on an Orbitrap Fusion Lumos mass spectrometer hyphenated to an easy-nLC 1200 nano HPLC system using a nanoFlex ion source (ThermoFisher Scientific, Waltham, MA, USA) which applied a spray voltage of 2.6 kV with the transfer tube heated to 300 °C and a funnel RF of 30%. Internal mass calibration was enabled (lock mass 445.12003 m/z). Peptide separation was performed on a self-made, 32 cm long, 75 µm ID fused-silica column, packed in house with 1.9 µm C18 particles (ReproSil-Pur, Dr. Maisch, Ammerbuch-Entringen, Germany) and heated to 50 °C in an integrated column oven (Sonation, Biberach, Germany).

Peptide fractions were eluted by a non-linear gradient from 9 to 32% B over 210 s with a step wise increase to 95% B in 16 s which was held for another 9 s. Full scan MS spectra (350–1400 m/z) were acquired with a resolution of 120,000 at *m*/*z* 200, maximum injection time of 100 ms and AGC target value of 4 ×105. We selected the 10 most intense precursors with a charge state between 2 and 5 per full scan for fragmentation and isolated with a quadrupole isolation window of 0.7 Th. MS2 scans were performed in the Ion trap (Turbo) using a maximum injection time of 85 ms, AGC target value of 2 × 104 and fragmented using CID with a normalized collision energy (NCE) of 35%. SPS-MS3 scans for quantification were performed on the 10 most intense MS2 fragment ions with an isolation window of 0.7 Th (MS) and 2 m/z (MS2). Ions were fragmented using HCD with an NCE of 65% and analyzed in the Orbitrap with a resolution of 50,000 at *m*/*z* 200, scan range of 110–500 *m*/*z*, AGC target value of 1 × 105 and a maximum injection time of 86 ms. Repeated sequencing of already acquired precursors was limited by setting a dynamic exclusion of 45 s and 7 ppm and advanced peak determination was deactivated.

### Mass spectrometry data analysis

Raw files were analyzed using MaxQuant 1.6 [45], with default settings using the Homo sapiens SwissProt database (TaxID:9606, version 2017-06-07). Gene ontology (GO) analysis was performed with DAVID 6.8 [46] using GOTERM_BP_DIRECT as a basis.

### Comet assay

The comet assay was performed according to the manufacturer’s instructions (ab238544, Abcam Plc., Cambridge, United Kingdom). Briefly, the LN-308 cells were seeded in six-well plates for 24 h, and then were treated with 400 µm temozolomide, 100 nM rapamycin and 1 mM NAC as indicated in serum-free medium for 24 h. The cells were washed PBS, and collected by gentle scraping. A total of 1500 cells in 15 μL were mixed with 60 μL agarose. The mixtures were transferred onto the provided comet slides and allowed to solidify at 4 °C for 30 min. The cells were lysed at 4 °C, and electrophoresis was performed with a cold alkaline electrophoresis buffer for 60 min. The comet slides were rinsed in H_2_O for 5 min three times, and were fixed with 70% cold ethanol for 5 min. The air-dried slides were stained with Vista Green DNA Dye and viewed using a fluorescence microscope. Percentage of DNA in the tail, tail length and tail moment was assessed using the ImageJ plugin OpenComet. A total of 50 nuclei were measured.

### Statistical analysis

Unless stated otherwise, all data is depicted as mean ± standard deviation (SD). Statistical analyses were performed with Microsoft Excel 2016 (Microsoft, Redmond, WA, USA) or Graph Pad Prism 9.2.0 (GraphPad Software, Inc., San Diego, CA, USA). The two-tailed Student’s *t*-test was used to determine *p* values. A value of *p* < 0.05 was considered to be statistically significant (*p < 0.05; **p < 0.01; ***p < 0.001). Calculation of sample size was conceptualized with 5% alpha error, 80% power and appropriate effect strength. Estimate of variant was not performed prior to any statistical analyses. The variance was similar in all comparison groups. Exclusion cirteria for samples were technical errors like spilling or pipetteing errors.

### Supplementary information

Original WesternBlot

Supplementary Figure legends

Supplementary Figure 1

Supplementary Figure 2

Supplementary Figure 3

Supplementary Figure 4

Supplementary Table 1

## Data Availability

The translatomic dataset generated during this study is included in this article and its supplementary information files. All other datasets used in the current study are available from the corresponding author on reasonable request.
