# Supplementary material for: Mammalian target of rapamycin inhibition protects glioma cells from temozolomide-induced cell death
Source: Cell Death Discov. 2024 Jan 5;10:8. doi: 10.1038/s41420-023-01779-2 (PMC10770336; doi:10.1038/s41420-023-01779-2)
Supplement: Supplementary file 2 — Supplementary Figure legends [file 41420_2023_1779_MOESM2_ESM.docx]

**Supplementary Figure 1.**

A, LN-308 cells were incubated in serum-free DMEM for 3 days with vehicle, 100 nM rapamycin, 100 nm Torin2 or 250 nm Torin 2 with or without 100 µM or 200 µm temozolomide. Cell density was assessed by CV staining (n=5, **p < 0.01 Student’s t-test) B, Corresponding vehicle control to Fig. 1D. LN-308, LNT-229 or G55T2 cells were incubated in serum-free DMEM for 3 days with vehicle in the presence of 100 nM rapamycin or 100 or 250 nM torin2. Cell death was assessed by PI staining (n=3, n.s. = not significant, Student’s t-test). C, Clonogenic survival assay of LN-308 and LNT-229 cells treated with vehicle, 10 µM temozolomide, 100 nM rapamycin or the combination (n=3, *p < 0.05, **p < 0.01 Student’s t-test).

**Supplementary Figure 2.**

LN-308 cells were treated as described in Fig 2. A, left panel: Volcano plot of fold changes versus p values between DMSO and rapamycin. Positive values display upregulated proteins in the combination group. The dashed line indicates a fold chance cutoff of 0.5 increase or decrease in translation, the red dots indicate p values < 0.05. Right panel: Bar chart of proteins significantly upregulated (blue) and downregulated (red) that cluster for gene ontology (GO). B, left panel: Volcano plot of fold changes versus p values between DMSO and temozolomide. Positive values display upregulated proteins in the combination group. Right panel: Bar chart showing the proteins significantly upregulated (blue) and downregulated (red) that cluster for gene ontology (GO).

**Supplementary Figure 3.**

Corresponding vehicle controls to Fig. 3 B-D. A-C, LNT-299, LN-308 and G55T2 cells were incubated serum free DMEM for 6 hours in the presence of vehicle, 100 nM rapamycin with and without the addition of 1 mM NAC. Cell density was assessed by CV staining (n = 5, n.s. = not significant, **p < 0.01 Student’s t-test). Cell death was assessed by PI staining (n = 3, n.s. = not significant, Student’s t-test).

**Supplementary Figure 4.**

LN-308 cells were incubated in the presence of vehicle, 400 µM temozolomide, 100 nM rapamycin or combinatory treatment with and without the addition of 10 µM BSO. A, Cell death was assessed by PI staining (n = 3, *p < 0.05, **p < 0.01 Student’s t-test). B, Cell density was assessed by CV staining (n=5, **p<0.01 Student’s t-test)
